# X-linked hypophosphatemic rickets and nephrocalcinosis: clinical characteristics of a single-center pediatric cohort in North America before and after burosumab

**DOI:** 10.3389/fped.2024.1430921

**Published:** 2024-08-02

**Authors:** Neil J. Paloian, Lindsey R. Boyke-Lohmann, Robert D. Steiner

**Affiliations:** ^1^ Department of Pediatrics, University of Wisconsin School of Medicine and Public Health, Madison, WI, United States; ^2^ Department of Orthopedics and Rehabilitation, University of Wisconsin School of Medicine and Public Health, Madison, WI, United States

**Keywords:** XLH, rickets, hypophosphatemia, nephrocalcinosis, burosumab

## Abstract

**Background:**

X-linked hypophosphatemic rickets (XLH) is a rare genetic disease characterized by inappropriately elevated circulating fibroblast growth factor 23 (FGF-23) and subsequent urinary phosphate wasting. The primary clinical manifestations of XLH include short stature, lower extremity bowing, dental abscesses, and rickets. Historical treatment includes phosphate and vitamin D supplementation, but recently, targeted therapy with burosumab has gained widespread acceptance. Burosumab is an FGF-23 blocking antibody. Conventional therapy options have been associated with the development of nephrocalcinosis (NC), with reported rates varying between 33% and 80% in XLH patients. Previous studies have noted that the phosphate supplementation dose correlates with the presence of NC, although this finding is not consistent across studies. It remains unclear whether nephrocalcinosis occurs in patients now treated with burosumab. Our aim was to identify XLH-associated nephrocalcinosis risk factors in our cohort of children with XLH and provide an updated analysis in the era of burosumab.

**Methods:**

We identified 13 children with XLH who received routine medical care for XLH at our institution between 2015 and 2023. All were initially treated with conventional therapy and were transitioned to burosumab either upon its US Food and Drug Administration (FDA) approval in 2018 or at 6 months of age if this occurred after 2018. All patients were routinely monitored and this included laboratory tests and renal ultrasonography. Phosphate and calcitriol dosages were regularly adjusted to minimize serum and urinary laboratory abnormalities. Burosumab was administered according to its FDA package insert directions. Medication doses and laboratory values were analyzed between the group with NC and the group without NC.

**Results:**

Three patients were noted to have evidence of NC within the study timeline. Two children developed NC while receiving conventional therapy and one while prescribed burosumab. None of the variables, including a positive family history of XLH, average age at diagnosis of XLH, duration or dosage of treatment with conventional therapy, average age at the initiation of burosumab, and all measured laboratory values, were significantly different between the groups with and without NC. Female sex was the only identified significant risk factor for a diagnosis of XLH-associated NC.

**Conclusion:**

XLH-associated NC remains a clinical concern even with modern treatment, although the traditional risk factors (dose of phosphate supplements and degree of urinary phosphate excretion) may not always correlate with the onset of nephrocalcinosis. XLH patients receiving burosumab, which has been hypothesized to eliminate the risk factors for NC, can still develop NC. It is important to continue screening patients treated with burosumab for nephrocalcinosis. In addition, more research is needed to better understand the risk factors that cause XLH-associated NC and determine whether children with XLH never exposed to conventional therapy will develop NC.

## Introduction

X-linked hypophosphatemic rickets (XLH) is the most common cause of hereditary rickets (1). Pathogenic variants in phosphate regulating endopeptidase X-linked (*PHEX*), the causative gene associated with XLH, lead to inappropriately elevated levels of fibroblast growth factor 23 (FGF-23) and subsequent urinary phosphate wasting (2). Clinically, XLH presents similarly to other forms of rickets, with childhood onset genu varum or genu valgum, and with a variable array of additional skeletal manifestations, including short stature, lower extremity bowing, craniotabes, craniosynostosis, wrist flaring, frontal bossing, and an expansion of the costochondral junction (3). The biochemical hallmark of XLH is significant hypophosphatemia due to excessive and inappropriate urinary phosphate excretion. Although children with XLH initially have normal calcium and parathyroid levels, over time, affected patients often develop hyperparathyroidism due to the role of FGF-23 as a downregulator of 1-α-hydroxylase activity and from therapeutic phosphate supplementation (4).

Historically, XLH has been treated with oral phosphate supplementation combined with vitamin D in the form of calcitriol (conventional therapy). XLH patients treated with conventional therapy demonstrate notably improved skeletal outcomes (5). However, up to 80% of children with XLH treated with conventional therapy develop nephrocalcinosis (NC), with the dose of phosphate supplementation being a suspected risk factor (6). Although the exact mechanism of development of nephrocalcinosis in XLH is not fully understood, it is believed that calcium phosphate intratubular calcifications form spontaneously in strong association with hyperphosphaturia (7). In addition, there is evidence that hypercalciuria, secondary to intermittent calcitriol-induced hypercalcemia, can exacerbate nephrocalcinosis in some patients (8). Most patients with XLH and nephrocalcinosis will have preserved renal function, but a subset of these patients, both children and adults, may develop chronic kidney disease over time secondary to nephrocalcinosis (9, 10). Therefore, XLH patients receiving conventional therapy require frequent monitoring of labs and medication dose adjustments to achieve amelioration of skeletal deformities without causing renal calcifications (1).

In April 2018, burosumab was approved by the US Food and Drug Administration (FDA) for the treatment of XLH in children and adults (11). Burosumab, a humanized monoclonal antibody targeting FGF-23, has been demonstrated to more effectively improve skeletal and biochemical abnormalities associated with XLH (12). In addition, by decreasing circulating FGF-23 levels, the use of burosumab lowers urinary phosphate excretion compared with conventional therapy in children with XLH; however, no published studies to date have assessed the prevalence of nephrocalcinosis in children with XLH treated with burosumab. Therefore, we aimed to examine the prevalence and risk factors of nephrocalcinosis in children with XLH prescribed conventional therapy or burosumab therapy.

## Methods

All individuals less than 21 years of age with a diagnosis of XLH and treated at the University of Wisconsin Hospitals and Clinics (UWHC) between 1 January 2015 and 31 December 2023 were included in this retrospective study. Inclusion criteria were clinical evidence of rickets with hypophosphatemia and an XLH diagnosis confirmed by the identification of a pathogenic variant in *PHEX*.

Before the introduction and approval of burosumab in 2018, all patients in our cohort were treated with conventional therapy (oral phosphate supplementation and calcitriol). In addition, following the introduction of burosumab, children less than 6 months of age at the time of XLH diagnosis were initially prescribed conventional therapy. Patients were initially prescribed 20–30 mg/kg of elemental phosphorous administered four to five times per day. Calcitriol was initially administered at a dose of 20 ng/kg given once or twice daily. The doses of phosphate and calcitriol were modified throughout the study period to attempt to correct hypophosphatemia and signs of rickets without producing hypercalciuria, hypercalcemia, or hyperparathyroidism, or significantly worsening hyperphosphaturia. In addition, the phosphate dose was frequently adjusted due to poor gastrointestinal tolerance (abdominal pain, cramping, and diarrhea). Laboratory testing was conducted approximately every 3 to 6 months with routine clinic visits. Blood work included serum phosphate (Phos) (mg/dl), calcium (Ca) (mg/dl), creatinine (Cr) (mg/dl), alkaline phosphate (U/L), and intact parathyroid hormone (PTH) (pg/ml). Hyperparathyroidism was defined as greater than the upper limit of our local institutional assay (>97 pg/ml). Urine testing included phosphate (mg/dl), calcium (mg/dl), and creatinine (mg/dl). Urinary calcium excretion was measured as Ca_urine_/Cr_urine_. Hypercalciuria was defined as a urine Ca/Cr above the upper limit of normal for age (13). Fractional excretion of phosphate (FE_Phos_) was calculated as (Phos_urine_/Phos_serum_) × (Cr_serum_/Cr_urine_) × 100. Renal tubular reabsorption of phosphate to glomerular filtration rate (TmP/GFR) was calculated as described previously (14). At every clinic visit, adherence to conventional therapy was assessed by parental or patient recall.

Burosumab was approved by the FDA in 2018, and after discussion with patients and their families, all patients were converted from conventional therapy to burosumab if they were over 6 months of age. Patients less than 6 months of age born on or after April 2018 were transitioned to burosumab at 6 months. In accordance with the manufacturer's instructions, phosphate supplementation and calcitriol were discontinued at least 7 days before starting burosumab. The dose of burosumab was selected based on the approved package insert; 1 mg/kg/dose rounded to the nearest 1 mg every 14 days in patients <10 kg, 0.8 mg/kg/dose rounded to the nearest 10 mg every 14 days in patients <18 years of age and >10 kg, and 1 mg/kg/dose rounded to the nearest 10 mg every 4 weeks for patients aged 18 years or older. As per the manufacturer’s instructions, the maximum single daily dose used was 90 mg. The decision to increase the burosumab dose in the setting of ongoing hypophosphatemia was at the discretion of the treatment team and the patient and their family. Burosumab doses were increased stepwise as per the manufacturer's instructions in these scenarios. If serum phosphate was noted to be greater than 5 mg/dl, burosumab was held and follow-up labs were obtained every 4 weeks until serum phosphate decreased to less than 5 mg/dl; at this point burosumab was restarted at the re-initiation dose as described in the manufacturer’s instructions. Adherence to burosumab therapy was assessed by a parental or patient report at clinic visits.

The same laboratory tests outlined above were obtained monthly for 3 months after the initiation of burosumab and with all dose changes. These labs were also initially obtained approximately every 3–6 months thereafter throughout treatment with burosumab and typically spaced out to annual lab testing after several years of stability.

Throughout the study period, renal ultrasonography was performed every 1–2 years regardless of treatment modality. Renal ultrasounds were interpreted by the Pediatric Radiology faculty members at the University of Wisconsin-Madison. A diagnosis of NC was determined if ultrasonography identified any increase in echogenicity of the medullary periods. The degree of nephrocalcinosis was graded as described previously (15). Briefly, grade 1 nephrocalcinosis corresponds with mildly increased echogenicity surrounding the perimeter of the medullary pyramids, grade 2 with moderately increased echogenicity around and starting to incorporate inside the pyramids, and grade 3 with severely increased echogenicity throughout the entirety of the medullary pyramids.

Statistical analyses were performed using a two-tailed Student's *t*-test and Microsoft Excel (Redmond, WA, USA). A *p*-value <0.05 was considered statistically significant. Approval for this study was granted by the University of Wisconsin Health Sciences Institutional Review Board.

## Results

Thirteen pediatric patients met the inclusion criteria. Baseline characteristics of these patients are detailed in Table 1. One patient turned 18 years old during the study period; the remaining 12 were all <18 years of age throughout the study timeline.

**Table 1:** Baseline characteristics of the XLH cohort.

	XLH patients
*N*	13
Age at XLH diagnosis [mean (SD), years]	1.5 (0.9)
Duration of conventional therapy [mean (SD), years]	4.2 (4)
Age at initiation of burosumab [mean (SD), years]	5.6 (5)
Female (%)	67
Positive family history of XLH (%)	85

**Figure 1:**
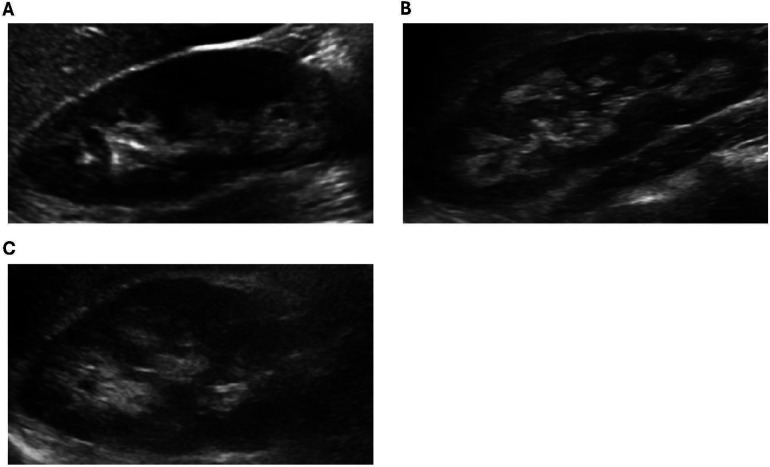
Representative ultrasound images from XLH study patients with NC. (**A**) Patient 1: increased echogenicity surrounding the medullary pyramids (grade 1). (**B**) Patient 2: increased echogenicity around and starting to incorporate inside the pyramids (grade 2). (**C**) Patient 3: increased echogenicity around and starting to incorporate inside the pyramids (grade 2).

Of the 13 XLH patients in our cohort, 3 were diagnosed with nephrocalcinosis during the study period. Further details regarding these patients are featured in Table 2. All three patients were female and had a family history of XLH. Typical images from our cohort with nephrocalcinosis are shown in Figure 1.

**Table 2:** Characteristics of patients with nephrocalcinosis.

	Patient 1	Patient 2	Patient 3
Age at diagnosis of XLH (years)	1	3	1
Age at diagnosis of NC (years)	12	7	7
Grade of NC	1	2	2
Years on conventional therapy at NC diagnosis	N/A	4	7
Total years on conventional therapy	7	7	18
Years on burosumab at NC diagnosis	5	N/A	N/A
Total years on burosumab therapy	6	6	6

Patient 1 was diagnosed with XLH at 1 year of age and started conventional therapy at that time. She was treated with conventional therapy for 6 years, at which point she was transitioned to burosumab. Routine ultrasonography during the course of conventional therapy noted no nephrocalcinosis. After 5 years of burosumab treatment, she was diagnosed with grade 1 NC. No further ultrasounds were obtained before the end of the study period.

Patient 2 was diagnosed with XLH at 3 years of age and started conventional therapy at 5 years of age. She was diagnosed with grade 2 NC after 4 years on conventional therapy, before starting burosumab. Five subsequent ultrasounds noted no change in the degree of NC. She was transitioned to burosumab at 9 years of age with no change in her NC noted while on burosumab.

Patient 3 was diagnosed with XLH at 1 year of age and started conventional therapy at this time. She was diagnosed with grade 2 NC after 7 years of conventional therapy, before starting burosumab. Seven subsequent ultrasounds noted no change in the degree of NC. She was transitioned to burosumab at 18 years of age with no change noted in her NC while on burosumab.

The three patients with NC were all female, whereas only 50% of the cohort without NC were female, which was a statistically significant difference. A positive family history of XLH, average age at XLH diagnosis, duration of treatment with conventional therapy, and average age at the initiation of burosumab were not statistically different between the two cohorts (Table 3).

**Table 3:** Baseline characteristics of patients with and without nephrocalcinosis.

	NC	No NC	*p*-value
*N*	3	10	n/a
Female (*N*, %)	3 (100)	5 (50)	0.01
Age at diagnosis [mean (SD), years]	1.7 (1.2)	1.5 (0.9)	0.84
Duration of conventional therapy [mean (SD), years]	10.7 (6.3)	4.1 (3.6)	0.2
Age starting burosumab [mean (SD), years]	10.2 (4.2)	2.6 (1.9)	0.07
Family history of XLH	3 (100)	8 (80)	0.17

Urinary mineral excretion did not differ significantly between the cohort with NC and the patients who did not develop NC. Average urinary calcium and phosphate excretion levels were not significantly different between the two groups. Although two patients without NC had hypercalciuria, no children with NC were found to have hypercalciuria at any point during the observational period. There were no significant differences in urine phosphate levels between the two groups measured with fractional excretion of phosphate and with TmP/GFR. Urinary mineral excretion is outlined further in Table 4. Two of the three patients with NC were diagnosed with hyperparathyroidism, whereas 2 of the 10 patients without NC were diagnosed with hyperparathyroidism; however, this was not a statistically significant difference.

**Table 4:** Laboratory values of patients with and without nephrocalcinosis.

	NC	No NC	*p*-value
*N*	3	10	n/a
Hypercalciuria (*N*, %)	0 (0)	2 (20)	0.17
Avg U_Ca/Cr_	0.05 (0.03)	0.05 (0.07)	0.96
Avg Fe_Phos_	14 (3)	19 (10)	0.25
Max Fe_Phos_	21 (8)	27 (23)	0.11
Avg TmP/GFR	2.61	2.88	0.39
Minimum TmP/GFR	1.77	1.81	0.86
History of PTH (*n*, %)	2 (66)	2 (20)	0.29

Doses of phosphate supplementation and calcitriol were examined between the group with NC and the group without NC. Neither the average phosphate dose nor the maximum phosphate dose were different between the two groups; although there was a trend toward higher phosphate dosing in the cohort with NC, this was not a statistically significant difference. In addition, the average calcitriol dose and the maximum calcitriol dose were not statistically different between the group that developed NC and the group without NC. Medication and supplement doses are further outlined in Table 5.

**Table 5:** Medication and supplement doses of patients with and without nephrocalcinosis.

	NC	No NC	*p*-value
*N*	3	10	n/a
Average phosphate dose (SD, mg/kg/day)	40 (18)	33 (11)	0.59
Maximum phosphate dose (SD, mg/kg/day)	51 (19)	42 (14)	0.49
Average calcitriol dose (SD, ng/kg/day)	17 (7)	16 (7)	0.85
Maximum calcitriol dose (SD, ng/kg/day)	21 (12)	18.4 (9.4)	0.75

Adherence to conventional therapy could not be quantified but was less than 100% based on interviews with parents at medical appointments. The reasons for this included difficulty with the frequent administration of phosphate and gastrointestinal side effects of the phosphate therapy. Adherence to burosumab therapy was reported to be 100% during the study timeline based on parental or patient recall at clinic visits. Except as listed above, no serious side effects were seen in any patient during treatment with either conventional therapy or burosumab.

## Discussion

NC is a known medical comorbidity of XLH, although the underlying pathophysiology of NC development is not well understood. A review of the available studies outlined in this discussion noted an inconsistency in risk factors that lead to NC in XLH patients. This current study helps explore the underlying mechanisms of NC development, including in the burosumab era.

It has long been suspected that the NC in XLH patients is a direct result of therapies used to treat XLH. Alon et al. identified two initial XLH patients who were found to have NC (16). Because of the small sample size, no conclusions could be drawn about the etiology of the NC, although it was noted that for high-risk patients like those with XLH, ultrasonography should be employed routinely for nephrocalcinosis screening. Subsequently Goodyer et al. studied 23 XLH patients and identified 11 with nephrocalcinosis and reported that the NC patients were prescribed higher doses of phosphate and higher doses of either vitamin D or activated vitamin D. This correlated with higher serum phosphate concentrations and more episodes of hypercalciuria in the NC group (8).

Reusz et al. studied 18 XLH patients and noted that six of them developed NC. The patients with NC had more episodes of hypercalciuria. In this study, there was a correlation with higher phosphate supplement doses and higher urinary phosphate levels in the patients with NC. This was suspected to be the primary cause of the hypercalciuria and resulting NC (17).

Kooh et al. subsequently examined 25 XLH patients. This cohort was found to have a prevalence of NC of 80%, which was not affected by the phosphate or calcitriol treatment dose (18). The frequency of NC observed was very similar to Verge et al. (1991) who discovered a 79% rate of NC in their XLH population of 24 patients. However, similar to Reusz et al. (1990), it was determined that the presence of NC was related to the dose of phosphate supplementation, with no difference in the vitamin D doses between the two groups. In addition, no biochemical differences were noted in the group with NC compared with the group without NC in the Verge et al. (1991) study population (6).

Keskin et al. (2015) followed six children with XLH, three of which developed NC. They noted a higher average dose of phosphate supplements in the NC group vs. the group without NC, but otherwise no differences in the doses of calcitriol or in any lab parameters (19). Most recently, Colares Neto et al. investigated 16 children with XLH and identified NC in 9 of them; adult XLH patients were also studied and had a lower frequency of NC. In this analysis, the children with XLH were prescribed higher doses of phosphate supplementation than the adult XLH patients and the authors hypothesized that this was due to the higher phosphate doses, although a direct comparison of phosphate doses in children with vs. without NC was not undertaken (9).

As expected, XLH patients with baseline urinary phosphate wasting exhibit an increase in urinary phosphate when oral phosphate supplementation successfully increases serum phosphate concentration. Alon et al. noted this pattern in five XLH patients with NC and in Hyp knockout mice fed a high phosphate diet. Further evidence of this was elicited when renal biopsies of the human patients and knockout mice revealed intratubular calcium phosphate depositions (7). As noted above, some previous studies have correlated this elevated phosphate loading in XLH patients with the development of NC. However, results over multiple studies have been inconsistent. This is likely due to many limiting factors, including small sample sizes, difficulty in measuring the compliance of the prescribed phosphate dosing, changes in the form of administered phosphate and vitamin D supplementation over time, and inconsistencies in accurately capturing phosphate doses over long periods of time due to the frequency of dose adjustments. Our study has demonstrated that phosphate supplementation doses do not always correlate well with the occurrence of NC in XLH patients. Although our sample size was similar to previous studies, we were able to accurately review the electronic medical record (something that would not have been possible for most of the older studies referenced). In addition, because of the reliability of capturing pharmacologic data with each dose change in the electronic health record, we could examine both the average dose over time and the maximum dose taken by our patients; this does add considerable strength to the accuracy of our results. Consistent with this, we also found no clear correlation with the presence of hypercalciuria or with increased urinary phosphate excretion as a risk factor for NC.

Our group has previously noted a significant decrease in urinary phosphate levels following a transition from conventional therapy to burosumab in children with XLH (12). Harada et al. examined urinary mineral excretion in a sample of XLH patients treated with burosumab and noted that their urinary metabolic profile improved. They hypothesized that XLH patients treated with burosumab should have protection against developing nephrocalcinosis (20). In our present study, we did identify a patient who developed NC while being treated with burosumab. This patient was previously treated with conventional therapy, although this treatment had been discontinued 5 years before the diagnosis of NC. This patient had two previous ultrasounds following the discontinuation of conventional therapy that were normal, confirming the time of the development of NC post conventional therapy. As this is the first patient that we are aware of to be diagnosed with NC while taking burosumab, no clear conclusions can be made regarding the pathophysiology of why she developed NC, especially in the context of a significant improvement in all laboratory and x-ray findings after transitioning to burosumab (12). However, this finding, at least until a better understanding is gained regarding the risk of NC with burosumab, confirms the need to continue performing screening ultrasounds on XLH patients treated with burosumab.

Interestingly, the only significant risk factor that we identified for the development of NC was female sex. Although XLH males have previously been identified as having NC, our finding is noteworthy as XLH is an X-linked genetic disease. Although females will have often have evidence of disease, their phenotype can be variable due to lyonization. Affected males, with only one X chromosome, will always be fully impacted. Therefore, it would be assumed that males would have a higher chance of developing nephrocalcinosis. It is presently unclear why female sex is a significant risk factor for XLH patients. By contrast, male sex (in the absence of XLH?) is associated with a higher chance of having urinary mineral abnormalities and kidney stone disease both in children and adults, although these differences may be changing over time (21, 22). Given these differences and the unique pathology of XLH, this is an area that deserves more research to obtain a full understanding of it.

Our current study does have several notable limitations. The primary limitation is the small sample size. Unfortunately, the small number of patients did not give us the power to perform the most robust statistical analysis; therefore, statistical methods were chosen that were in line with previous XLH cohort studies. Given the status of XLH as a rare disease, it is difficult to amass a large cohort of XLH patients. Although our population is relatively large for this rare disease, it is still small overall; larger more diverse XLH populations are needed to better understand all the risk factors for XLH-associated nephrocalcinosis. This study (like all the others referenced above) is a single-center retrospective study. Single institutions may care for patients of similar ethnic or socioeconomic backgrounds. In addition, patients treated at one medical center are likely to have little variation in provider management; this is especially true in regard to conventional therapy. Finally, at a specialty clinic like ours, significant attention was paid throughout the study to adjusting phosphate doses to prevent urine mineral levels from becoming excessively abnormal; this bias could have certainly impacted our total doses of phosphate and calcitriol administered (although we still had three patients develop NC despite this). The impact of these factors requires evaluation on a much larger scale with multicenter studies. These data are currently being collected via the XLH-DMP (disease monitoring program) REF. This international data registry is prospective and includes children and adults diagnosed with XLH. Some data elements will be difficult to fully capture, such as primary recall of past phosphate doses; this study will hopefully help to clarify clear-cut risk factors for NC development in XLH patients.

The risk factors of XLH-associated nephrocalcinosis remain unclear as studies have reported conflicting and inconsistent results. Most studies have described the primary risk factor as the administered phosphate supplementation dose; however, several studies, including this current study, have not been able to reproduce this. We have identified one patient who developed NC years after stopping phosphate supplementation, which would argue against phosphate supplementation and ongoing hyperphosphaturia as the main risk factor for developing XLH-related NC. Given these results, it is likely that the pathophysiology of XLH-associated NC is much more complex than excessive urinary phosphate driven by high doses of phosphate supplements, as previously believed. In the current analysis, the only risk factor that was significantly associated with NC was female sex, underscoring the need for more research to better understand the pathology and mechanisms that lead to nephrocalcinosis in XLH patients, especially females. Larger prospective likely multicenter studies that include adults and children are needed to better comprehend the physiologic mechanisms that may lead to increased nephrocalcinosis in females with XLH and fully capture the risk of nephrocalcinosis in these patients treated with burosumab. Furthermore, if burosumab treatment is subsequently approved for use from birth or if some clinicians prescribe it off label, it will be important to study whether NC occurs in XLH individuals who are never exposed to therapy with phosphate and calcitriol.

## Data Availability

The original contributions presented in the study are included in the article/Supplementary Material, further inquiries can be directed to the corresponding author.
