# Erratum: X-linked hypophosphatemic rickets and nephrocalcinosis: clinical characteriscs of a single-center pediatric cohort in North America before and after burosumab

**DOI:** 10.3389/fped.2024.1514803

**Published:** 2024-11-05

**Authors:** Frontiers Production Office

**Affiliations:** Frontiers Media SA, Lausanne, Switzerland

**Keywords:** XLH, rickets, hypophosphatemia, nephrocalcinosis, burosumab

An Erratum on X-linked hypophosphatemic rickets and nephrocalcinosis: clinical characteristics of a single-center pediatric cohort in North America before and after burosumab By Paloian NJ, Boyke-Lohmann LR and Steiner RD (2024). Front. Pediatr. 12: 1430921. doi: 10.3389/fped.2024.1430921

Due to an error, the funder for the article processing charge “Dr. Penniston (Department of Urology, University of Wisconsin-Madison)” was erroneously omitted.

The conflict of interest statement was incorrectly reported as “RS reports consulting fees from Leadiant, Mirum, PTC, and Travere. He is a shareholder of Exact Sciences. The remaining authors declare that the research was conducted in the absence of any commercial or financial relationship that could be construed as a potential conflict of interest”.

The correct conflict of interest statement is “RS reports consulting fees from Leadiant, Mirum, PTC, and Travere. He is a shareholder of Exact Sciences. NJ, LB-L, RS, and the funder, Dr. Penniston, are all employed by the University of Wisconsin”.

The publisher apologizes for this mistake. The original version of this article has been updated.

